# The relationship between serum uric acid and lipid profile in Bangladeshi adults

**DOI:** 10.1186/s12872-019-1026-2

**Published:** 2019-02-21

**Authors:** Nurshad Ali, Sadaqur Rahman, Shiful Islam, Tangigul Haque, Noyan Hossain Molla, Abu Hasan Sumon, Rahanuma Raihanu Kathak, Md Asaduzzaman, Farjana Islam, Nayan Chandra Mohanto, Mohammad Abul Hasnat, Shaikh Mirja Nurunnabi, Shamim Ahmed

**Affiliations:** Department of Biochemistry and Molecular Biology, Shahjalal University of Science and Technology, Sylhet, 3114 Bangladesh

**Keywords:** Serum uric acid, Dyslipidemia, Cardiovascular disease, Adults, Bangladesh

## Abstract

**Background:**

Although the link between elevated uric acid and metabolic syndrome has been reported in some studies; the relationship of serum uric acid (SUA) with lipid profile has not well studied or little is known so far. This study was conducted to assess the relationship between SUA and lipid profile among the general adults in Bangladesh.

**Methods:**

In total, 280 blood samples were collected from general adult participants (male, *n* = 150 and female, *n* = 130) and analyzed for serum lipid profile (TC, TG, HDL and LDL) and SUA levels. The study subjects were divided by quartiles based on SUA levels (Q1: ≤225 μmol/L, Q2: 226–285 μmol/L, Q3: 286–340 μmol/L and Q4: > 340 μmol/L). Linear regression modeling was used to evaluate the relationship between SUA and lipid levels.

**Results:**

The prevalence of hyperuricemia was 9.2% in males and 10.4% in females. The mean level of SUA was significantly higher in male (317 ± 90 μmol/L) than in the female (255 ± 65 μmol/L) subjects (*p* < 0.001). An increasing trend for elevated lipid profile was observed in both gender with increasing levels of SUA in the quartiles (*p* < 0.05). In regression analysis, a significant positive correlation was found between SUA and TG, TC and LDL (*p* < 0.01) while an inverse correlation was observed between SUA and HDL (*p* < 0.01). After adjusting for potential confounders, lipid profile was linearly associated with SUA levels (*p* < 0.01 for trend).

**Conclusions:**

Present study showed a significant positive relationship for SUA with TG, TC and LDL levels, and an inverse relationship for SUA with HDL. Early prevention of hyperuricemia and dyslipidemia may be helpful to reduce the incidence of associated cardiovascular diseases.

## Background

In humans, serum uric acid (SUA) is the final oxidation product of purine catabolism [1]. Excessive uric acid production and its decreased excretion by the kidneys are one of the major causes of hyperuricemia [2, 3]. The prevalence of hyperuricemia is rapidly increasing in the international communities; emerging evidence shows that hyperuicemia is now more frequent in the developing nations [4]. The variability in SUA levels is multi-factorial and influenced by both genetic and environmental factors [5]. Epidemiological studies showed that elevated levels of uric acid in serum are increasingly related to hypertension, cardiovascular disease (CVD) and metabolic syndrome [6–8]. In previous studies, SUA concentrations were higher in individuals with coronary heart disease than in healthy subjects and elevated SUA was found to be associated with increased cardiovascular morbidity and mortality in the general adult population [9, 10].

Hyperuricemia is considered to be a mediator of proinflammatory endocrine imbalance in the adipose tissue which may be one of the important factors for dyslipidemia and the inflammatory process that leads to atherogenesis [11]. The relationship of uric acid with CVD risk factors has made it very complicated to determine whether uric acid has a causal role in these conditions or simply a marker for individuals at increased risk, reflecting the association with other traditional risk factors such as blood lipids, metabolic syndrome and diabetes [12–14]. The exact role of SUA in these diseases is still the debate and subject of much discussion because it is always accompanied by other risk factors such as diet, dyslipidemia and obesity [15]. Moreover, the relationship between SUA and dyslipidemia is complex and not fully elucidated yet [15]. A few studies have been conducted to investigate the association between SUA and lipid profiles in the adult population of India [12], Italy [9] and USA [15]. However, still, there is a lack of information on the association of SUA with lipid profile for the Bangladeshi adult population. In this study, we aimed to assess the independent relationship between SUA and lipid profile in a Bangladeshi adult cohort.

## Methods

### Study design and participants

This study was a cross-sectional design, conducted between September and December of 2017. The study consisted of 280 general adult participants (150 males and 130 females), recruited from university staffs, university students, city people and individuals who went to the local clinic for the routine health check-up in Sylhet and Dhaka city regions of Bangladesh. All the participants were apparently healthy individuals without any severe cardiovascular diseases. Informed consent was obtained from all participants’ prior to inclusion in the study. This study was approved by the internal Ethics Committee of the Department of Biochemistry and Molecular Biology, Shahjalal University of Science and Technology, Bangladesh. Participants with myeloproliferative disorders and in therapy with cytotoxic drugs, pregnant women, lactating mothers and the individuals who are already on the diuretic, anti-hypertensive, hypolipidemic, alcoholics, known cardiovascular disorders, renal or hepatic disorders and those on anti-gout therapy were excluded from the study.

### Anthropometric measurements and blood sample collection

Anthropometric indices of height, weight, waist and hip circumference and other lifestyle information were obtained using the standard procedure described elsewhere [16, 17]. Height was measured to the nearest 0.1 cm and weight was measured to the nearest 0.1 kg by modern electronic digital LCD weighing machines (Beurer 700, Germany) wearing light clothing and no shoes. The scales were calibrated everyday against a standard (20 kg). The body mass index (BMI) was calculated as weight (kg) divided height squared (m^2^). Waist circumference (WC) was measured midway between the lowest border of the ribs and iliac crest in the horizontal plane and hip circumference (HC) was measured at a level parallel to the floor, at the largest circumference of the buttocks to the nearest 0.5 cm with anthropometric tape. The quality of anthropometric measurements was ensured in presence of investigators.

### SUA and lipids measurements

Venous blood (5 mL) was drawn from each participant under strict aseptic precautions and allows clotting at room temperature and centrifuged at 3000 rpm for 15 min. Serum was separated for analysis of biochemical parameters. Serum uric acid (SUA), and serum lipids: triglycerides (TG), total cholesterol (TC), high-density lipoprotein (HDL) and low-density lipoprotein (LDL) were analyzed by colorimetric methods using commercially available kits (Human Diagnostic, Germany). All measurements were done according to the manufacturer’s protocols (Human Diagnostic, Germany) with a semi-auto biochemistry analyzer (Humalyzer 3000, USA). The measurements were done by trained staff and the accuracy of the analysis was confirmed through standard calibration on regular basis.

### Diagnostic criteria

In the present study, participants were classified as hyperuricemic with SUA levels > 416.4 μmol/L in men and > 356.9 μmol/L in women [18, 19]. The levels of SUA were categorized into four quarterlies based on frequencies test. According to the National Cholesterol Education Programme guideline the optimal level of TC, TG, HDL and LDL were < 200 mg/dl, < 150 mg/dl, > 40 mg/dl and < 100 mg/dl, respectively [20].

### Statistical analysis

Statistical analysis was performed using IBM SPSS version 23. Independent sample t-test (two-tailed) was done to assess the differences between male and female cohort for anthropometric and baseline variables. Interrelationships between anthropometric, baseline variables and SUA were assessed by Pearson’s correlation coefficient test. One-way ANOVA was performed to determine differences among the groups. The linear regression modeling was applied to evaluate the association between SUA quartiles and lipid levels. Three models were used with progressive degrees of adjustment. Model 1 was adjusted for age, gender, BMI. Model 2 was further adjusted for age, gender, BMI and WC. Model 3 was adjusted for age, gender, BMI, waist and hip circumference. The values in tables were presented as mean ± standard deviation otherwise noted. A *p* value of < 0.05 was considered statistically significant.

## Results

### Demographic characteristics of the study cohorts

The baseline characteristics of the study subjects are presented in Table 1. Among a total of 280 participants, 150 (54%) were males and 130 (46%) were females. The mean age was 32 ± 12 years (range 18–75 years), with a significant difference between male and female subjects (*p* < 0.01). The average BMI for all participants was 24 ± 4 kg/m^2^ with no significant difference between the gender groups. The mean value of WC was 85 ± 7 with a significant difference between male and female (*p* < 0.05) subjects. A significant difference was also observed for the average levels of SUA, TG and HDL in the gender groups. Based on diagnostic criteria, overall the prevalence of hyperuricemia was 9.8% among the participants with 9.2% in male and 10.4% in female subjects.

**Table 1:** Baseline characteristics and SUA level according to gender

	Overall	Male	Female	*P*-value
*N*	280	150 (54%)	130 (46%)	–
Age (years)	32 ± 12 (75)	35 ± 14 (75)	30 ± 10 (60)	0.007
Height (cm)	158 ± 8 (176)	166 ± 5 (176)	152 ± 5 (165)	0.000
Weight (kg)	63 ± 10 (90)	67 ± 9 (85)	59 ± 10 (90)	0.000
WC (cm)	85 ± 7 (115)	86 ± 8 (104)	82 ± 8 (115)	0.046
HC (cm)	94 ± 8 (122)	93 ± 6 (105)	94 ± 10 (122)	0.078
BMI (kg/m^2^)	24 ± 4 (36)	25 ± 3 (33)	25 ± 4 (36)	0.298
SUA (μmol/L)	290 ± 85 (505)	317 ± 90 (505)	255 ± 65 (440)	0.000
Hyperuricemia (%)	9.8	9.2	10.4	0.288
TG (mg/dl)	152 ± 88 (373)	170 ± 90 (360)	130 ± 84 (373)	0.004
TC (mg/dl)	137 ± 48 (256)	130 ± 54 (256)	144 ± 40 (252)	0.065
HDL (mg/dl)	44 ± 12 (82)	40 ± 10 (64)	48 ± 15 (82)	0.000
LDL (mg/dl)	75 ± 39 (210)	70 ± 40 (210)	82 ± 35 (188)	0.110

Results are presented as mean ± SD with maximum values in parentheses. *P*-values are given for differences between the gender groups

### SUA quartiles and comparison of lipid profile in the quartiles

The characteristics of the study participants by SUA quartiles are summarized in Table 2. The individuals with higher SUA quartiles were more likely to be male participants. After adjustment of age and sex, the mean level of SUA, TG, TC and LDL were progressively increased and HDL level was progressively decreased across the SUA quartiles. According to the national cholesterol education guideline, the percentage of dyslipidemic risk among the subjects was progressively increased in the SUA quartiles.

**Table 2:** Characteristics of the study population by SUA quartiles

SUA levels (μmol/L)						
	Overall	Q1 (≤225)	Q2 (226–285)	Q3 (286–340)	Q4 (> 340)	*p*-value
*N*	280	69	72	72	67	–
Gender (m/f)	150/130	23/46	34/38	43/29	50/17	–
Age (years)	32 ± 13	34 ± 14	31 ± 13	33 ± 12	31 ± 12	0.302
BMI (kg/m^2^)	25 ± 4	24 ± 4	25 ± 4	26 ± 4	26 ± 3	0.003
WC (cm)	84 ± 8	80 ± 9	83 ± 10	87 ± 7	88 ± 6	0.002
HC (cm)	94 ± 7	90 ± 6	94 ± 7	96 ± 6	97 ± 7	0.004
SUA (μmol/L)	296 ± 21	192 ± 25	258 ± 15	325 ± 12	410 ± 30	0.000
TG (mg/dl)	156 ± 85	135 ± 82	130 ± 70	175 ± 105	184 ± 84	0.005
% of risk (TG)	26	20	25	30	31	–
TC (mg/dl)	139 ± 47	125 ± 45	129 ± 48	146 ± 48	156 ± 47	0.035
% of risk (TC)	18	12	15	21	22	–
HDL (mg/dl)	43 ± 12	46 ± 13	44 ± 14	43 ± 11	39 ± 10	0.040
% of risk (HDL)	41	34	40	45	46	–
LDL (mg/dl)	76 ± 38	67 ± 36	68 ± 43	82 ± 35	88 ± 42	0.045
% of risk (LDL)	30	24	30	30	35	–

Values are presented as mean ± SD. P-values are obtained from one way ANOVA

^*^Risk values of serum lipids: total cholesterol > 200 mg/dl, triglycerides > 200 mg/dl, HDL cholesterol < 40 mg/dl, LDL cholesterol > 100 mg/dl [19]

### Association of SUA with lipid profile

**Fig. 1:**
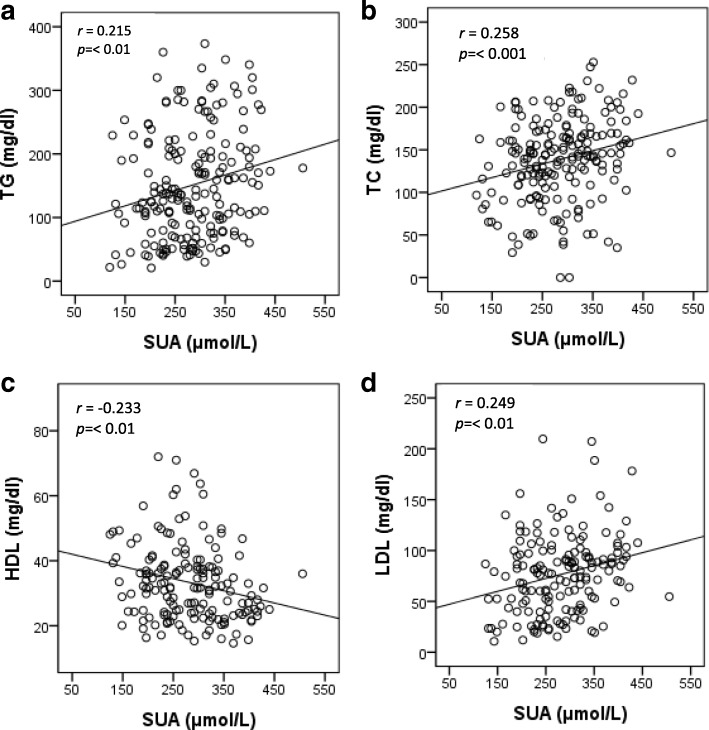
Association between SUA and TG (**a**), TC (**b**), HDL (**c**) and LDL (**d**). The scale in the Y-axis is not similar in all figures

A statistically significant positive association (*p* < 0.01) was observed for serum uric acid levels with serum TG, TC and LDL levels, where as a significant negative association was found between serum uric acid serum HDL level (Fig. 1). After adjusting for age and gender (model 1), serum TG, TC and LDL levels in individuals in the highest quartile of serum uric acid levels were higher than in the lowest quartile (p for trend < 0.01). Serum HDL cholesterol in the highest quartile of SUA levels was lower than in the lowest quartile (p for trend < 0.01). The correlation remained unchanged after additionally adjusting for other covariates in model 2 and 3 (Table 3).

**Table 3:** Association of SUA quartiles with TG, TC, HDL, LDL and TG to HDL ratio

SUA level (μmol/L)					
	Q1 (≤225)	Q2 (226–285)	Q3 (286–340)	Q4 (> 340)	*P* for trend
TG					
Model 1	0.00 (Ref.)	0.19 (0.16, 0.24)	0.29 (0.25, 0.34)	0.46 (0.42, 0.51)	< 0.01
Model 2	0.00 (Ref.)	0.13 (0.08, 0.18)	0.14 (0.06, 0.20)	0.26 (0.21, 0.34)	< 0.01
Model 3	0.00 (Ref.)	0.13 (0.08, 0.18)	0.14 (0.04, 0.18)	0.27 (0.22, 0.35)	< 0.01
TC					
Model 1	0.00 (Ref.)	0.23 (0.18, 0.28)	0.34 (0.28, 0.38)	0.45 (0.38, 0.50)	< 0.001
Model 2	0.00 (Ref.)	0.19 (0.12, 0.26)	0.21 (0.12, 0.30)	0.26 (0.15, 0.35)	< 0.001
Model 3	0.00 (Ref.)	0.17 (0.08, 0.24)	0.19 (0.11, 0.28)	0.24 (0.16, 0.34)	< 0.001
HDL					
Model 1	0.00 (Ref.)	−0.05 (−0.05, −0.03)	−0.09 (−0.08, −0.06)	− 0.13 (− 0.14, − 0.10)	< 0.01
Model 2	0.00 (Ref.)	− 0.04 (− 0.04, − 0.01)	− 0.06 (− 0.07, − 0.04)	−0.08 (− 0.12, − 0.06)	< 0.01
Model 3	0.00 (Ref.)	− 0.04 (− 0.03, − 0.01)	−0.06 (− 0.06, − 0.03)	−0.05 (− 0.10, − 0.04)	< 0.05
LDL					
Model 1	0.00 (Ref.)	0.12 (0.05, 0.18)	0.22 (0.14, 0.30)	0.30 (0.24, 0.38)	< 0.01
Model 2	0.00 (Ref.)	0.10 (0.04, 0.20)	0.15 (0.05, 0.28)	0.24 (0.10, 0.36)	< 0.01
Model 3	0.00 (Ref.)	0.10 (0.02, 0.20)	0.15 (0.04, 0.26)	0.22 (0.08, 0.34)	< 0.01
TG to HDL ratio					
Model 1	0.00 (Ref.)	0.18 (0.13, 0.24)	0.28 (0.22, 0.34)	0.54 (0.44, 0.56)	< 0.01
Model 2	0.00 (Ref.)	0.10 (0.04, 0.18)	0.12 (0.06, 0.20)	0.30 (0.20, 0.38)	< 0.01
Model 3	0.00 (Ref.)	0.10 (0.01, 0.16)	0.10 (0.01, 0.18)	0.32 (0.22, 0.40)	< 0.01

Adjusted covariates: model 1 = age, gender, and BMI; model 2 = age, gender, BMI and WC, model 3 = age, gender, BMI, WC and HC

## Discussion

The present study was conducted to assess whether hyperuricemia without a known CVD is associated with increased lipid levels so that identifying and treating such individual can prevent the development of CVD. To the best of our knowledge, this is the first study that reports the strong association between SUA and lipid profile for the Bangladeshi adults. Two important implications can be drawn from the present study. First, SUA levels were positively associated with serum TG, TC, LDL cholesterol and the ratio of TG to HDL cholesterol. Second, there was an inverse association between SUA and HDL cholesterol level regardless of adjustment for gender and several potential confounders, indicating a crucial role of uric acid in the regulation of dyslipidemia. These findings are in line with previous studies that showed a pathogenesis overlap among hyperuricemia and dyslipidemia [9, 15, 21].

A number of risk factors are associated with CVD, which can be grouped into modifiable and non-modifiable. Atherogenic dyslipidemia, including high TG, and LDL cholesterol levels with low HDL cholesterol levels is a modifiable risk factor in humans [22]. The association of atherogenic dyslipidemia to cardiovascular risk has been reported in previous epidemiological studies [23, 24]. The link of between hyperuricemia and CVD has been established in several studies [10, 25, 26]. Hyperuricemia predisposes to the development of hypertension and may increase the oxidative stress and generate of free radicals, which eventually can be the source of future cardiovascular disease [11]. Although it still needs to be investigated whether the observed relationship between increased SUA and CVD is a causative or simply epidemiological; several lines of evidence report that determination of uric acid in serum or plasma might be helpful in early predict the risk of CVD [9]. In present study, LDL cholesterol showed a linear correlation with SUA even after adjusting co-variants. A similar finding has been observed in a recent study [15]. In this study, the TG to HDL ratio, a known indicator of insulin resistance, showed a positive association with SUA as reported in a previous study [27]. Previous Studies, also, demonstrate that hyperuricemia can affect adipocytes by increasing monocyte chemoattractrant protein and reducing the production of adiponectin, thereby contributing to insulin resistance and inflammation [11, 28]. These finding indicated a complex interaction between SUA and lipids which remains unclear. Taking into account present study results, we are agreed with a previous study remarks that uric acid may intensify several pathophysiological mechanisms that are associated with the CVD risk and may have synergistic interaction with other lipid profile causing CVD [15].

Serum HDL cholesterol is a known protective factor for CVD risk. In our study, serum HDL cholesterol was inversely correlated with SUA which is in line with the findings of previous studies [9, 15]. The elevated levels of SUA have been considered a significant predictor of smaller and denser of LDL and HDL particles, which offers a greater atherogenic ability [29]. The lower levels of HDL cholesterol favors the formation of atherosclerosis and eventually predisposed to CVD, although the direct evidence of the positive role of HDL in reducing CVD has not clearly understood yet [15]. A linear correlation was found between TG and SUA in some previous studies [21, 30] which are also in line with the results of present study. It is assumed that the synthesis of TG requires NADPH, which resulted in increased SUA production [15].

The concurrence of dyslipidemia and hyperuricemia has been reported in a few studies. For example, a significant association was found between SUA and lipid profile in the adult population of India [12], Italy [9] and USA [15]. In recent years, the prevalence of hyperuricemia has been predisposed by the increasing frequency of several risk factors, such as obesity, hypertension and metabolic syndrome [31]. These observed associations influenced each other by diverse mechanism and precipitated by a number of factors. Therefore, it is important to develop proper treatment guidelines counting diet, lifestyle modification, and pharmacologic measures to reduce hyperuricemia and its adverse health effects. Moreover, reduction of SUA needs to be considered since this strategy may act synergistically with lipid-lowering therapies to reduce the cardiovascular risk [32]. The limitations of present study are: first, the cross-sectional nature of the data may preclude the cause-effect relationships between SUA levels and lipid profile being assumed. Second, relatively a small sample size which may not represent the observed findings for the entire population of Bangladesh. Third, we did not have individual food habits information which may affect lipid levels. However, present study findings are worthy as a reference. A prospective longitudinal study considering the association between prior dyslipidemia and incident hyperuricemia would be valuable to confirm the observed association.

## Conclusion

The present study shows a strong association between SUA and lipid profile among the Bangladeshi adults. Early prevention of hyperuricemia and dyslipidemia can reduce the incidence of associated cardiovascular disease among the Bangladeshi adults. Further, investigations are needed taking into account of hypertension, diabetes, and lifestyle for a better understanding of the observed association.

## Abbreviations

BMI: Body mass index
CVD: Cardiovascular disease
HDL: High-density lipoprotein
LDL: Low-density lipoprotein
TC: Total cholesterol
TG: Triglycerides
